# Cryptococcal antigen among HIV1-infected individuals in north-central Nigeria

**DOI:** 10.18502/CMM.6.2.3662

**Published:** 2020-06

**Authors:** Chimezie Ezenabike, Oluwaseyi S. Ashaka, Adesuyi A. Omoare, Abayomi Fadeyi, Alakija K. Salami, Olajide O. Agbede

**Affiliations:** 1 Department of Medical Microbiology and Parasitology, College of Health Sciences, University of Ilorin, Ilorin, Nigeria; 2 Department of Medicine, College of Health Sciences, University of Ilorin, Ilorin, Nigeria

**Keywords:** CD4+ T-cell count, Cryptococcosis, HIV-1, Lateral flow assay

## Abstract

**Background and Purpose:**

The potential for the invasion of the central nervous system by *Cryptococcus* species is underscored by the presence of this organism in the blood of immunocompromised individuals. Early adoption of sensitive methods for the diagnosis of *Cryptococcus* species will reduce the high morbidity and mortality associated with this disease. Regarding this, the aim of the present research was to detect cryptococcal antigen among HIV1- infected individuals in north-central Nigeria.

**Materials and Methods:**

This prospective cross-sectional study was carried out on HIV-1 infected individuals accessing care at three health facilities in north-central Nigeria between November 2014 and March 2017. For the purpose of the study, blood samples were collected from 300 HIV1-infected individuals within the age group of 3-65 years. The CD4+ T-cell count was determined, and the samples were analyzed for cryptococcal antigenemia using the methods of lateral flow assay (LFA) and culture technique

**Results:**

*Cryptococcus* antigen was detected in 19.67% (59/300) of the patients, and only 25.4% (15/59) of the LFA-positive samples showed *Cryptococcus* species growth on Sabouraud dextrose agar after 3 days. Furthermore, fungal growth was observed in one of the specimens, which was LFA negative. Additionally, 30 of the 59 LFA-positive patients had cryptococcal antigen in their serum with a CD4+ T-cell count of < 150 cells/mm^3^

**Conclusion:**

As the findings of the present study indicated, infection with *Cryptococcus* species is a problem among HIV-infected patients in the region under study. Therefore, all HIV patients, especially those with a CD4+ T-cell count of < 150 cells/mm^3^, referring to the HAART clinics in Nigeria, should be screened for cryptococcal antigen

## Introduction

cryptococcosis received attention at the inception of HIV/AIDS era. Based on the statistics, Sub-Saharan Africa has the highest annual mortality due to cryptococcosis in HIV patients [ 1]. As estimated, up to 504,000 deaths annually occur as a result of cryptococcal meningitis. This figure has been maintained despite the available antiretroviral and antifungal therapies [ 1]. The fungi accounting for the development of this disease enter the body through the respiratory tract. After some days, they are disseminated and can be detected in the cerebrospinal fluid where they cause meningoencephalitis [ 2]. 

*Cryptococcus* species is able to invade the central nervous system (CNS) and synthesizes melanin from catecholamines, which are present at large concentrations in the CNS. Melanin has antioxidant properties, protecting the organism from oxygen-dependent immune actions [ 3]. The potential for CNS cryptococcal meningitis is underscored by the untimely presence of infection in the immunocompromised individuals. The HIV-infected individuals and organ transplant recipients are among the groups who are at the risk of cryptococcal meningitis. Other populations at risk include children, pregnant women, and those living in resource-limited environments [ 4]. 

*Cryptococcus neoformans* infection has become a critically relevant opportunistic infection as a consequence of the HIV/AIDS pandemic [ 5]. Diagnosis of cryptococcal meningitis requires the implementation of the cerebrospinal fluid (CSF) culture of Cryptococcus species, microscopic examination with Indian ink staining, or CrAg testing in the CSF or serum [ 6]. Serum cryptococcal antigen (CrAg) is recognized as a marker for an invasive or disseminated cryptococcal infection, as well as the most sensitive and specific indicator for systemic cryptococcosis [ 7]. It is known that CrAg can be detected in blood some weeks or months prior to the development of overt clinical symptoms. Therefore, the screening of CrAg in serum provides an opportunity to identify people with an asymptomatic disease at an early stage [ 6, 8].

There is a report regarding the unsuccessful management of two cases of cryptococcosis among HIV patients at the University of Ilorin Teaching Hospital, Ilorin, Nigeria. This failure is an indication of the extent of *C. neoformans* infection fatality in HIV1-infected individuals with a low CD4+ T-cell count in case of the unavailability of the recommended antifungal therapy [ 9, 10 ]. Therefore, the aim of this study was to detect cryptococcal antigen in HIV1-infected individuals in north-central Nigeria where about 2% of the population are living with HIV [ 11].

## Materials and Methods

***Study design***

This prospective cross-sectional study was carried out among HIV1-infected individuals accessing care at three health facilities in north-central Nigeria between November 2014 and March 2017. It was aimed to identify the risk neglected during screening despite the reported cases of cryptococcosis-induced deaths. This study was carried out at the Federal Medical Center and Kogi State Specialist Hospital, Lokoja, and University of Ilorin Teaching Hospital, Ilorin, Nigeria. The study population was selected using the purposive sampling as a non-probability sampling method.

**Study population**

Blood samples of 300 HIV1-infected individuals were taken and investigated for detecting cryptococcal antigen and culturing. Prior to the study, written informed consent was obtained from the adult patients and parents/guardians of children. Finally, the subjects who had not used any antifungal medication in the last 72 h before the study were recruited from three hospital facilities in north-central Nigeria. On the other hand, the patients who had not been on highly active antiretroviral therapy for more than a month were excluded from the study. 

**Ethical Considerations**

The current study was conducted in compliance with the Helsinki Declaration and approved by the Health Research and Ethics Committee of Federal Medical Centre, Lokoja, Kogi State Specialist Hospital and University of Ilorin Teaching Hospital, Ilorin (ERC PAN/2015/10/1459). All data were analyzed anonymously throughout the study. A semi-structured questionnaire was administered to obtain relevant information on sociodemographic characteristics and laboratory findings.

***Sample Collection***

For the purpose of the study, 5 mL blood was drawn from each participant by a phlebotomist and labeled appropriately. Subsequently, aliquots were decanted into tubes without anticoagulant for serum extraction to detect cryptococcal antigen, as well as for blood culture. They were also poured into EDTA anticoagulant tubes to determine the CD4+ T-cell count.

***Cryptococcal Antigen Detection using Lateral Flow Assay***

Detection of cryptococcal antigen was accomplished using the lateral flow assay (LFA) by means of the Immy Latex-Crypto Antigen kit (Immuno-Mycologics, Inc., Norman, Oklahoma). This method involves the impregnation of gold-conjugated, monoclonal antibodies onto an immunochromatographic test strip to detect cryptococcal capsular polysaccharide glucuronoxy-lomannan antigen for all four *C. neoformans* serotypes (A-D) [ 12]. Comparison of the LFA assay with the gold standard diagnostic method of cryptococcosis (culture) showed that the test had the sensitivity and specificity of 100% for serum and CSF antigen. Regarding the plasma antigen, these values were obtained as 98.9% and 100%, respectively. This assay was performed according to the manufacturer’s written instructions. 

***Blood Culture and Species Identification***

The isolation of *Cryptococcus* species was carried out using Sabouraud dextrose agar (Becton Dickinson and Company, USA). To this end, the aliquots of whole blood were collected into tubes without anticoagulant and centrifuged at 3,000 rpm for 10 min. Subsequently, a drop of the serum was spread on agar slants in the three tubes of Sabouraud-chloramphenicol medium and incubated at 30°C for up to 3 weeks. The isolates were subcultured in Sabouraud dextrose agar slants in screw-top glass test tubes to confirm purity. *Cryptococcus* species was identified by urease hydrolysis; furthermore, the Indian ink preparation revealed that each isolate was encapsulated [ 13, 14]. 

Cultures, yielding smooth, cream-buff colored, moist, and mucoid colonies at 37°C, were suspected to be positive. Microscopy results showed a circular stained core and an outer unstained halo, on a dark background. Encapsulated yeast cells were seen as refractile bodies and surrounded by the unstained thickness of the capsule. The characteristic pinched-off budding confirmed *Cryptococcus* species. *Streptococcus pneumonia* ATCC 49619 and *Candida albicans* ATCC 10231 were also used as positive and negative controls, respectively. 

**CD4 + T-cell count**

The CD4+ T-cell count in whole blood was determined using flow cytometry (Partec Cyflow SL–3 Münster, Germany) according to the manufacturer’s written instructions.

**Statistical analysis**

The obtained results are presented in tables. The data were entered into the computer and analyzed with Epi Info (version 7.1.43.) developed by the Centers for Disease Control and Prevention (USA; website at http://www.cdc.gov/epiinfo). This was used to generate the frequencies and measures of central tendencies. Correlation of the categorical variables was evaluated using the Chi-square test. A p-value less than 0.05 was considered statistically significant. 

## Results 

A total of 300 HIV-infected individuals within the age group of 3- 65 years participated in this study. The mean age of the participants was 39.6±19.7 years. The majority of the subjects were adults (35-44 years) as shown in Table 1. Regarding gender distribution, out of 300 participants, 180 cases were female. Cryptococcal antigen was detected in 59 (19.7%) HIV-infected individuals, including 31 females (52.5%) and 28 males (47.5%; χ^2^=1.702, *P*=0.1920). Only 16 (5.3%) patients had blood cultures which showed growth, identified as *Cryptococcus* species. These cases consisted of 6 (37.5%) females and 10 (62.5%) males that showed the evidence of fungal growth by culture (χ^2^=3.565; *P*=0.0590).

**Table 1:** Comparison of cryptococcal antigen detection and blood culture techniques.

	Culture positive (%)	Culture negative (%)	Total
**LFA positive**	15 (25.4)	44 (74.6)	59
**LFA negative**	1 (0.4)	240 (99.6)	241
**Total**	16 (5.3)	284 (94.7)	300

Sensitivity: 93.75%, specificity: 84.51%, likelihood ratio =6.051, χ^2^=58.71, *P*<0.0001.

LFA: Lateral Flow Assay.

**Figure 1:**
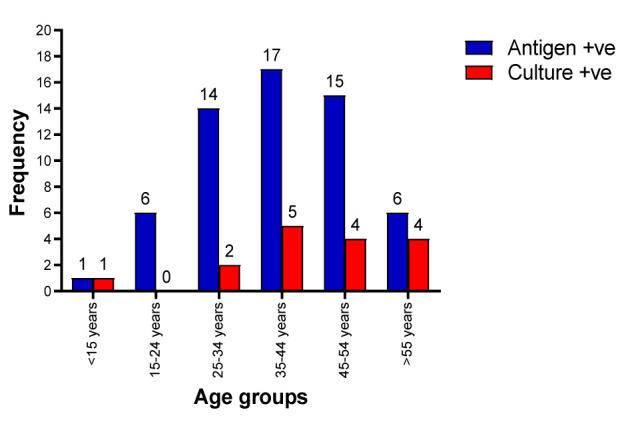
Frequency of *Cryptococcosis* by blood culture and antigen detection among HIV1-positive individuals.

Comparison of cryptococcal antigen detection and culture among different age groups showed that the age group of 35-44 years had the highest rate of Cryptococcus species detection based on blood culture and antigen. In this regard, 5 cases were found to be positive by culture, and 17 subjects had positive cryptococcal antigen. On the other hand, the age group of < 15 years had the lowest rate of *cryptococcal* antigen detection (n=1). Additionally, no fungal growth was observed among the age group of 15-24 years (Figure 1). There was a statistically significant difference when CrAg detection was compared with blood culture among different age groups (t=2.634, df=10; *P*=0.0250).

A total of 15 participants were truly positive because they had cryptococcal antigen in their serum and showed the evidence of fungal growth by culture. It should be mentioned that 44 (approximately 75%) patients had false-positive results because there was no observed fungal growth by culture, even when cryptococcal antigen was detected in their serum. Only one participant had a false-negative result when *Cryptococcus* antigen was assayed but had the evidence of fungal growth by culture as shown in Table 1. The sensitivity and specificity of cryptococcal antigen test were 93.75% and 84.51%, respectively. In this regard, the CrAg test had a six-time likelihood of detection in comparison with culture (likelihood ratio=6.051, χ^2^=58.71; *P*<0.0001).

The CD4+ T-cell count of the participants ranged from 104 to 1,152 cells/mm^3^. A total of 41 (13.67%) participants had a CD4+ T-cell count of ≤ 150 cells/mm^3^. The majority (70.6%) of this group had cryptococcal antigen in their serum. In addition, more than 25% of the cases with a CD4+ T-cell count of ≤ 150 cells/mm^3^ showed the evidence of Cryptococcus species by culture as shown in Table 2. Based on the results, the CD4+ T-cell count was significantly different between the cases with a cell count of < 150 cells/mm^3^ and those with a cell count of > 150 cells/mm^3^ based on both antigen detection and culture techniques (cryptococcal antigen: χ^2^=78.38, *P*<0.0001; blood culture: χ^2^=43.46, *P*<0.0001).

**Table2:** Prevalence of cryptococcal antigen detection and blood culture in terms of CD4+ T-cell count.

CD4+ T-cell count	Cryptococcal antigen		Culture		
	Positive (%)	Negative (%)	Positive (%)	Negative (%)	Total
≤150	29 (70.7)	12 (29.3)	11 (26.8)	30 (73.2)	41
>150	30 (11.6)	229 (88.4)	5 (1.9)	254 (98.1)	259
Total	59 (19.7)	241 (80.3)	16 (5.3)	284 (94.7)	300

Cryptococcal antigen: χ^2^=78.38, *P*<0.0001; Blood culture: χ^2^=43.46, *P*<0.0001.

## Discussion

Cryptococcal antigens can be detected in the serum several weeks before the onset of the symptoms. Accordingly, the patients who are asymptomatic but positive are at a heightened risk of mortality as a result of cryptococcal meningitis [ 8 ]. Based on the results of the present study, CrAg had a prevalence of 19.7% among the HIV-infected individuals in north-central Nigeria. This implies the presence of a high cryptococcal disease burden among HIV-infected individuals. This may be the result of exposure to *C. neoformans* from birds or the environment. 

Chukwuanukwu *et al*. [ 15] suggested that the presence of *Cryptococcus* species in HIV could be the result of the reactivation of latent infection in the event of immunosuppression. In other studies performed in Nigeria, the evidence of *Cryptococcus* antigen among HIV-infected patients was reported to have the prevalence of 2.2-13.1%, which is lower than the prevalence obtained in this study [ 15, 19]. The different methods used in these studies could account for the variation in the obtained results.

In another study carried out in Jos, north-central Nigeria, *C. neoformans* was reported to contribute about 36.0% to the burden of meningitis among HIV patients in the region under study [ 20]. The results of the present research showed a higher prevalence of cryptococcal meningitis in Nigeria because the conventional fungal culture of CSF samples was used as the gold standard for the diagnosis of cerebrospinal meningitis. Furthermore, other studies, which were performed in Nigeria that used CrAg to test HIV patients regardless of gender and CD4+ T-cell count, obtained the prevalence rates of 5.1% and 9.8% in Calabar and Benin City, respectively [ 17 , 19 ]. The number of screened participants and the sample selection criteria in the mentioned studies contributed to the reported prevalence. 

Mamoojee *et al*. [ 21] in Ghana recorded a lower prevalence (2%), compared to the prevalence recorded in our study. The sample size used in the two studies is not similar; however, the criterion for specimen selection in the Ghanian study was based on a CD4+ T-cell count of < 100 cells ⁄ mm^3^, which is representative of advanced HIV disease. Furthermore, Beyene et al. [ 14] reported a cryptococcal antigenemia prevalence of 20.9% in Ethiopia among HIV-infected patients with a CD4+ T-cell count of ≤ 150 cells/ mm^3^, which is similar to the prevalence obtained in this research. The obtained prevalence in these studies suggests the contribution of some other risk factors to the incidence of cryptococcal antigenemia among these patients.

Females had a higher rate of CrAg detected in their blood when compared to their male counterparts. This may be the result of the recruitment of more females than males in the study population. However, there was no statistically significant difference between CrAg detection among the male and female genders in this study. This is similar to the observation made by Osazuwa *et al*. [ 18 ], who found a higher level of *cryptococcal* antigen among the females. On the contrary, when blood culture results were compared among males and females in this study, males had a higher level of Cryptococcus species in their serum, compared to females. Nonetheless, this difference was not statistically significant. Likewise, Dzoyem *et al*. [ 13 ] reported a higher level of *C. neoformans* isolates in males than in females.

In the current study, although CrAg was detected in all the age groups under investigation, the age group of 35-44 years had the highest prevalence of CrAg detection and culture. This implies the exposure of all age groups in the population to this infection. In other studies, the age group of > 25 years has been reported to have the peak prevalence of CrAg [ 18, 19]. However, these studies could not justify the relationship between age and cryptococcal antigen detection.

In the present study, out of the 59 patients with cryptococcal antigenemia, there was evidence of the growth of *C. neoformans* in 15 patients using blood culture. Our study presented evidence regarding the growth of *Cryptococcus* species in an extraneural site due to the increased fungal load in the patients [ 13]. The sensitivity and specificity of cryptococcal antigen detection were respectively 93.75% and 84.51%, compared to those of culture (likelihood ratio=6.051, χ^2^=58.71, *P*<0.0001). The difference in the obtained sensitivity and specificity could be the result of the method used to characterize *C. neoformans*. 

Based on the findings, the CrAg test had a six-time likelihood of detection in comparison with culture. This is in line with the WHO guidelines and PEPFAR technical guidance that CrAg screening should be immediately implemented in the individuals presenting with low CD4 counts [ 22, 23]. The results of another study, comparing antigen detection with culture, demonstrated the sensitivity and specificity of 100% and 96.42%, respectively. The observation made in the mentioned study was different from what was obtained in the present study. However, the method of antigen detection applied in the mentioned study was latex agglutination [ 24].

Several studies that evaluated the prevalence of serum CrAg among HIV-infected individuals reported a higher prevalence in patients with a lower CD4+ T-cell count [ 18]. In this study, cryptococcal antigen was detected in the majority of the patients with a CD4+ T-cell count of ≤ 150 cells/mm^3^. This can be associated with the number of patients who had a CD4+ T-cell count of < 200 cells/mm^3^ post-recruitment. Joseph *et al*. [ 17] reported a prevalence of 13.5% among the participants with a CD4+ T-cell count of < 100 cells/mm^3^.

The results of the present study were suggestive of the high burden of cryptococcal antigenemia among HIV-infected individuals in north central Nigeria. Several countries in Sub-Saharan Africa have implemented CrAg screening to mitigate the effects of cryptococcal meningitis in HIV-infected population. Data of this study are indicative of the necessity of CrAg testing among the immunosuppressed HIV population in Nigeria. The present study also entails a number of limitations. We did not follow up the participants with antigenemia to determine the proportion of individuals who had secondary CNS or pulmonary infection. Furthermore, the presence of *C. neoformans* was not confirmed by means of sensitive molecular techniques, such as polymerase chain reaction. 

## Conclusion

The results of this study revealed the likelihood of the progression of cryptococcal disease among HIV-infected individuals with a CD4+ T-cell count of < 150 cells/mm^3^ with cryptococcal antigenemia in the area under investigation. Therefore, it is required to perform the routine screening of CrAg test for both children and adults attending HIV clinics in north-central Nigeria, as well as across the country. Furthermore, the provision of adequate antifungal regimen for the treatment of those with cryptoccocal antigenemia will facilitate early intervention and better management of cryptococcal meningitis cases. 
